# Comparative Analysis of Metabolites of ‘Hongro’ Apple Greasiness in Response to Temperature

**DOI:** 10.3390/foods12224088

**Published:** 2023-11-10

**Authors:** Hyang Lan Eum, Ji-Hyun Lee, Me-Hea Park, Min-Sun Chang, Pue Hee Park, Jae Han Cho

**Affiliations:** Postharvest Technology Division, National Institute of Horticultural and Herbal Science, Rural Development Administration, Wanju 55365, Republic of Korea; leejh80@korea.kr (J.-H.L.); poemmich@korea.kr (M.-H.P.); aeru@korea.kr (M.-S.C.); puehee@korea.kr (P.H.P.); limitcho@korea.kr (J.H.C.)

**Keywords:** ‘Hongro’, ‘Fuji’, greasiness, temperature, ethylene, fatty acid, ester, terpene

## Abstract

Greasiness in apple skin reduces its quality, and its level varies depending on the variety. In this study, low-temperature (1 ± 0.5 °C) stored ‘Hongro’ and ‘Fuji’, which had differences in the occurrence of greasiness, were moved to room temperature (20 °C) and untargeted metabolite and fatty acids for skin and flesh along with quality changes due to greasiness occurrence were compared. Ethylene production differed noticeably between the two varieties and increased rapidly in ‘Hongro’ until 9 d of room-temperature storage. The ethylene production did not differ significantly between the two varieties on day 20 when greasiness occurred. According to the PLS-DA score plot, while ‘Hongro’ had similar amounts of unsaturated and saturated fatty acids, ‘Fuji’ had approximately twice as much unsaturated-fatty-acid content. ‘Hongro’, after 50 d of low-temperature (1 ± 0.5 °C) storage, produced excessive ethylene during room-temperature storage, which was directly related to greasiness development. As a result, the primary wax components of greasy ‘Hongro’ were nonacosane and nonacosan-10-ol. As the room-temperature storage period elapsed, pentyl linoleate and α-farnesene contents increased significantly. Furthermore, these greasiness-triggering characteristics of ‘Hongro’ may have been genetically influenced by the paternal parent used during breeding.

## 1. Introduction

The National Institute of Horticultural and Herbal Science in Korea selected ‘Hongro’ apples (*Malus* × *domestica* Borkh) in 1988 by crossing and cultivating ‘Spur Earliblaze’ and ‘Spur Golden Delicious’. ‘Hongro’ hold the largest market share before the late-flowering variety ‘Fuji’ (Red Delicious × Virginia Ralls Genet) is shipped. With a soluble solids concentration (SSC) of 14–15 °Bx and titratable acidity of 0.2–0.3%, ‘Hongro’ is popular among consumers due to its excellent taste and texture [1]. However, at room temperature, many lipid substances are generated in ‘Hongro’ fruit skin, resulting in a sticky texture when touched. Some consumers perceive that it has been chemically treated, causing a decrease in the intention to purchase ‘Hongro’ [2].

Cuticle, a hydrophobic layer composed primarily of cuticular waxes and cutin, is synthesized in the epidermis of horticultural crops and covers the entire epidermis [3,4,5,6]. Cuticular wax is the plant’s outermost layer and is composed of intracuticular and epicuticular wax. While intracuticular wax is embedded in the cutin polymer matrix, epicuticular wax exists on the cutin polymer’s outer layer, preventing plant water loss and protecting agricultural products from various external environments [3,6]. The majority of apple cuticular wax constituents are aliphatic compounds and triterpenes; aliphatic compounds include long-chain alkanes, alcohols, aldehydes, fatty acids, and ketones [7]. In addition, the main triterpene in apples is ursolic acid, which is found in the skin approximately four times more than in the flesh [8].

Changes in the physical and chemical properties of waxy substances found in apple skin significantly affect the apple’s appearance [5,9], which directly relates to the criteria consumers use to purchase apples. Greasiness occurs more frequently as the period of exposure to room temperature increases, and it is reported to occur frequently in ‘Cripps Pink’, ‘Janagold’, ‘Royal Gala’ and ‘Granny Smith’ varieties [5,10]. Greasiness occurrence is associated with farnesol accumulation, a type of acyclic sesquiterpenoid composed of 15 carbons [11], and is highly correlated with an increase in ethylene concentration [2,12]. Greasiness occurrence was reduced through treatment with 1-methylcyclopropene (1-MCP), an ethylene action inhibitor, and aminoethoxyvinylglycine, which inhibits the activity of aminocyclopropane-1-carboxylic acid synthase [2,10]. Ethylene increases during the ripening process of climacteric-type fruits such as apples and has a significant impact on quality and physiological mechanisms such as reduction in firmness, increase in soluble sugars, and formation of aroma components [13,14,15]. Apples, which can be stored for a long time, are a commodity that produces much ethylene. Additionally, exposure to external ethylene promotes senescence and reduces quality by producing more endogenous ethylene [16]. However, when stored at low temperatures, ethylene production is reduced, and the activity of various enzymes is reduced, thereby delaying physiological changes related to ripening and senescence [16,17].

Among the varieties grown in Korea, greasiness occurs more frequently in ‘Hongro’ grown domestically, and the amount of greasiness increases primarily when distributed at room temperature. This is a significant issue raised by ‘Hongro’ consumers and distributors. In this study, we investigated greasiness occurrence during storage at low and room temperatures for ‘Hongro’ and ‘Fuji’ apples, which differ in the amount of greasiness. Among them, we examined changes in wax composition in ‘Hongro’, which affects the development of greasiness depending on the storage period at low temperatures.

## 2. Materials and Methods

### 2.1. Plant Material

Apples (*Malus domestica* Borkh.) grown on a farm in Gunwui (36.23° N, 128.44° E), Gyeongsangbuk-do, Republic of Korea, were used to compare the quality of ‘Hongro’ and ‘Fuji’ during storage at room temperature. ‘Hongo’ apples (*n* = 100) were harvested in September 2020, and ‘Fuji’ apples were harvested in October. The apples were brought to the National Institute of Horticultural and Herbal Science laboratory, and were classified into apples of a specific size and without physical damage. We selected 100 apples each of ‘Hongro’ and ‘Fuji’. ‘Hongro’ and ’Fuji’ apples were stored at low temperature (1 ± 0.5 °C, 90–95% RH) for 2 months and 1 month, respectively, and then moved to room temperature (20 °C, 70–80% RH). Apples brought to room temperature were stored for 20 days, and their quality was evaluated. Apples harvested in September 2021 in the same area were used for an experiment to determine the effect of greasiness on the storage properties of ‘Hongro’ during storage at room temperature. The harvested apples were transported using a reefer truck (8–10 °C, 75–85% RH) to the laboratory and selected. We selected 200 apples without mechanical damage, and the fruit was divided into two groups, 100 per group. One group was stored at room temperature (20 °C, 70–80% RH) for 20 days, and the rest were stored at low temperature (1 ± 0.5 °C, 90–95% RH). Apples stored at low temperature for 50 days moved to room temperature, and the quality evaluation was conducted for 20 days

### 2.2. Quality Assessment

First, apples stored at room and low temperature were observed for weight loss, ethylene production, respiration rate, color index (CIE; lightness, L*; green to red, a*; blue to yellow, b*; saturation, chroma; and hue angle), and firmness. Second, weight-loss rate (%), soluble solids content (SSC), titratable acidity (TA), color index (CIE L*, a*, b*, chroma, hue angle), and firmness were measured to assess quality changes during storage. Weight change during storage was expressed as a percentage of the initial weight loss. After juicing the flesh, SSC was measured using a refractometer (PR-101; Atago, Tokyo, Japan) and expressed as °Bx. TA was measured using a pH meter (FiveEasy Plus; Mettler Toledo, Columbus, OH, USA), converted to malic acid, and expressed as %. Color was measured using a colorimeter (CR 400; Minolta, Osaka, Japan) at three locations in the equatorial region of the apple skin, and firmness (N) was measured after removing the skin in the fruit equatorial region with a peeler using a 120 mm/s, 10 mm probe. Firmness was measured using a texture analyzer (TA Plus LLOYD Instruments; Ametek, Berwyn, PA, USA).

Carbon dioxide and ethylene production were measured during storage by placing one fruit in a 1 L airtight container, sealing it for 1 h at 20 °C, and measuring the gas accumulated inside using a gas chromatograph (Agilent 6890A GC System; Agilent Technologies, Santa Clara, CA, USA). Carbon dioxide and ethylene were measured using a thermal-conductivity detector (column: 100 °C, detector: 120 °C, carrier gas: He (30 mL/min)) and a flame-ionization detector (column: 150 °C, detector: 180 °C, carrier gas: He (30 mL/min)), respectively, and a packed stainless-steel column. Carbon dioxide and ethylene standard gases were purchased from Hankook Special Gases Company (Iksan, Jeonbuk, Republic of Korea).

### 2.3. Assessment of Skin Greasiness

Skin greasiness was assessed as described by Yang et al. [5] with some modifications. The greasiness score (GS) was quantified by rubbing the apple’s skin by hand, and was classified into four grades: 0 ≤ GS ≤ 1, no greasiness; 2 ≤ GS ≤ 4, slight greasiness; 5 ≤ GS≤ 7, moderate greasiness; and 8 ≤ GS ≤ 10, severe greasiness. The panel’s acceptable range for greasiness was within GS 4.

### 2.4. Total Wax Content and Epicuticular Wax Quantification

Cuticular wax was extracted as previously described [5] with some modifications. Total cuticular-wax content was measured using apples stored for 10 and 20 d at room and low temperatures. One apple was immersed in 400 mL of chloroform for 1 min to extract wax. This process was repeated thrice; all solvents were mixed, and foreign substances were removed by filtering through Miracloth. The apple’s surface area was calculated using the ImageJ program. The solvent was concentrated using a nitrogen evaporator (EvaN-0600, Goojung, Seoul, Republic of Korea) at 30 °C and thoroughly dried to determine the total wax content. The dried sample was stored at −70 °C until GC/MS analysis of epicuticular wax. Chloroform (5 mL) containing 100 mg L^−1^ n-tetracosane (internal standard) was added to the frozen extracted wax. The extract (0.3 mL) was transferred to another vial, and the solvent was evaporated using a nitrogen evaporator and derivatized by adding 150 μL of bis-N,N-(trimethylsilyl) trifluoroacetamide (BSTFA) containing 1% trimethylchlorosilane (TMCS; Sigma-Aldrich, St. Louis, MO, USA) mixture. The vial was incubated at 75 °C for 70 min and then subjected to GCMS (GCMS-QP 2020 NX; Shimadzu, Kyoto, Japan). The oven temperature was initially maintained at 150 °C for 1 min, then increased to 300 °C at 12 °C min^−1^, and maintained for 7 min. The injector and detector temperatures were set to 270 °C. The flow rate of helium carrier gas was 1.2 mL min^−1^ and was measured using a DB–5 (Agilent; 30 m, 0.25 mm, 0.25 μm) capillary column. The mass scan range was 40 to 650. Compound identification was based on the NIST library, and a standard mixture of saturated alkanes from C7 to C40 (Supelco, Bellefonte, PA, USA) and hexacosanol were used as standards. Quantification of wax compounds was expressed as equivalent concentration using standard alkanes or hexacosanol.

### 2.5. Fatty-Acid Analysis

Fatty acids in apple peel and pulp were analyzed using the method of Rafael and Mancha [18] with some modifications. Mix 2 mL of methylation mixture (MeOH:Benzene:DMP(2,2-Dimethoxy-propane):H_2_SO_4_ = 39:20:5:2) and 1 mL of heptane with freeze-dried flesh or skin tissue (0.01 g). Afterward, it was extracted at 80 °C for 2 h. After extraction, when cooled at room temperature, it separated into two layers, and the supernatant was taken and analyzed using a Agilent 7890A GC system (Agilent Technologies, Wilmington, DE, USA). GC conditions were injector temperature 250 °C and programmed initial temperature 50 °C maintained for 1 min; it subsequently increased to 130 °C with 25 °C min^−1^, then to 170 °C with 8 °C min^−1^, and to 215 °C with 2 °C min^−1^, and was maintained for 10 min, and followed by 10 °C min^−1^, increasing to 250 °C and kept for 1 min. A flame-ionization-detector temperature was 280 °C with an air-flow rate of 350 mL min^−1^, and a hydrogen-gas-flow rate of 35 mL min^−1^ with a DB-23-fused silica capillary column (0.25 mm × 60 m × 0.25 μm; Agilent, Wilmington, DE, USA). Identification was based on injection of supelco 37 component FAME Mix (Supelco, Bellefonte, PA, USA) prepared from pure standards.

### 2.6. Untargeted Polar-Phase-Metabolite Analysis

Polar phase metabolites were studied using methods adapted from Hyun et al. [19] and Lee et al. [20]. The freeze-dried sample (0.05 g) was mixed with 1 mL of 80% methanol and sonicated for 30 min at 60–70 °C. After extraction, centrifugation was performed at 4 °C and 15,000× *g* for 10 min, and 700 μL of the supernatant was collected. The supernatant was mixed with 500 μL chloroform, 20 μL ribitol (internal standard), and 700 μL DW; vortexed; and centrifuged at 2500× *g* for 10 min. At room temperature, 500 μL of the supernatant was concentrated under reduced pressure using a nitrogen evaporator (EvaT-0200; Goojung Engineering Co., Seoul, Republic of Korea). Then, 50 μL methoxyamine hydrochloride (10 mg/mL in pyridine) was added to the concentrated sample, reeluted, and reacted in the dark at 37 °C for 2 h. After the reaction, 40 μL of the sample was reacted with 100 μL N-methyl-N-trifluoroacetamide in a vial in the dark at 37 °C for 30 min, and the GC-MS ISQ LT system (Thermo Fisher Scientific, Waltham, MA, USA) was used while increasing the oven temperature from 50 °C to 310 °C at 5 °C min^−1^. The injector was set to splitless mode at 250 °C. Helium was used as a carrier gas at a flow rate of 1 mL min^−1^ with a DB-5-fused silica capillary column (0.25 mm × 30 m × 0.25 μm; Agilent). The mass scan range was 35–550 *m/z*.

### 2.7. Statistical Analysis

A completely randomized design was used. Measurements and analyses were performed in triplicate. The data were reported as the mean ± standard deviation. ANOVA was performed using SAS (version 9.1, Cary, NC, USA). The significance of each measurement was determined using Duncan’s multiple range test at a significance level of *p* < 0.05. To investigate the relationship between groups, we used Metabonalyst 5.0 (www.metaboanalyst.ca, accessed on 14 October 2023) to obtain a general outline of metabolites among samples.

## 3. Results and Discussion

### 3.1. Physiological Response during Storage of ‘Hongro’ and ‘Fuji’ Showing Differences in Greasiness

**Figure 1:**
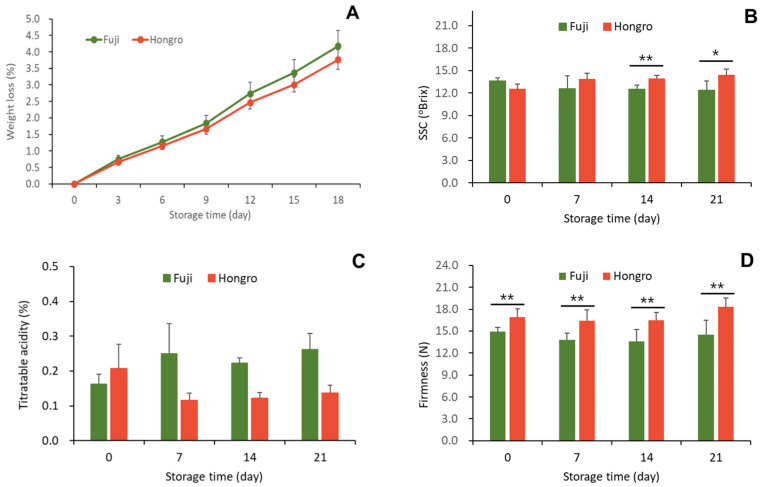
Changes in weight loss (**A**), SSC (**B**), titratable acidity (**C**), and firmness (**D**) of apples (‘Hongro’ vs. ‘Fuji’) during storage at room temperature (20 °C). Values are expressed as the means ± standard deviations. * represents significant differences, * *p* < 0.05, and ** *p* < 0.01. (*n* = 6).

By comparing the physiological responses of ‘Hongro’ and ‘Fuji’, the cause of the difference in greasiness occurrence between the two breeds was identified. The weight-loss rate of ‘Hongro’ and ‘Fuji’ stored at 20 °C for 3 weeks was approximately 4%, with no significant difference between the varieties (Figure 1A). SSC was slightly higher in ‘Hongro’ during storage, but there was no difference between varieties after one week. After 2 weeks of storage, the SSC of ‘Hongro’ was significantly high (*p* < 0.01) and remained high until 3 weeks of storage (*p* < 0.05) (Figure 1B). ‘Fuji’ tended to have a slightly higher acidity than ‘Hongro’, and this trend showed a significant difference until 3 weeks had passed (*p* < 0.001) (Figure 1C). ‘Hongro’ had a significantly higher firmness than ‘Fuji’ (*p* < 0.01), but there was no significant difference depending on the storage period for each variety (Figure 1D). The greasiness on ‘Hongro’’s skin appeared after approximately 20 d at 20 °C.

Changes in the color index of the pericarp were observed over 3 weeks of storage at room temperature. There was no significant difference in CIE L* and CIE b* values between the two varieties during the storage period (Figure S1a,b). The CIE a* value did not differ during storage for either variety; however, it did differ between varieties (Figure S1c). ‘Fuji’ maintained the range of 22–24, and ‘Hongro’ maintained the range of 26–28. This difference in CIE a* value affected the chroma value and hue angle, and ‘Hongro’ tended to have a higher saturation (*p* < 0.05) and lower hue angle value (*p* < 0.05) than ‘Fuji’ (Figure S1d,e).

**Figure 2:**
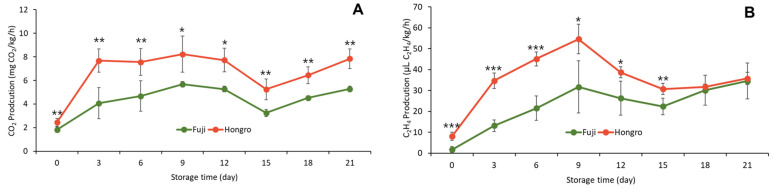
Changes in CO_2_ production (**A**) and C_2_H_4_ production (**B**) of apples (‘Hongro’ vs. ‘Fuji’) during storage at room temperature (20 °C). Values are expressed as the means ± standard deviations. * represents significant differences, * *p* < 0.05, ** *p* < 0.01, and *** *p* < 0.001. (*n* = 6).

In ‘Hongro’, where greasiness occurred, the CIE L* value would increase slightly after 20 d of storage; however, DMRT revealed no significant difference depending on the storage period (Figure S1a). Apple is a climacteric fruit [21], and a climacteric peak appeared during storage at room temperature. Furthermore, ‘Hongro’ demonstrated higher respiration and ethylene production than ‘Fuji’ (Figure 2A).

According to a previous study, apples with greasiness produced more ethylene than those without greasiness. Treatment with 1-MCP reduced greasiness. Ethylene has been linked to greasiness and affects wax composition [2,10,22].

Ethylene production in ‘Hongro’ was significantly higher than in ‘Fuji’. Until 9 d of storage, ethylene levels increased rapidly (Figure 2B). The ethylene levels did not differ significantly with greasiness between the two varieties during days 18–20 at room temperature, suggesting that ethylene caused greasiness symptoms to appear as the storage period elapsed.

**Figure 3:**
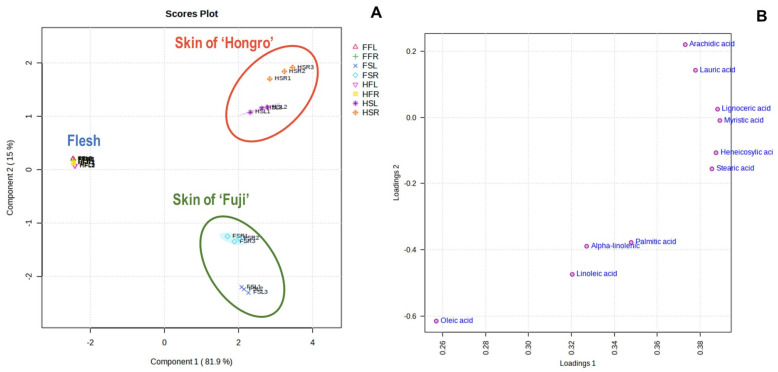
Comparison of fatty acids between ‘Hongro’ and ‘Fuji’ apples. (**A**) Score plot and (**B**) loading plot obtained using partial least squares discriminant analysis of fatty acids of skin and flesh of the apple varieties. FFL, ‘Fuji’ flesh stored at low temperatures for 1 month; FFR, ‘Fuji’ flesh stored at room temperature for 20 d; FSL, ‘Fuji’ skin stored at low temperatures for 1 month; FSR, ‘Fuji’ skin stored at room temperature for 20 d; HFL, ‘Hongro’ flesh stored at low temperatures for 1 month; HFR, ‘Hongro’ flesh stored at room temperature for 20 d; HSL, ‘Hongro’ skin stored at low temperatures for 1 month; and HSR, ‘Hongro’ skin stored at room temperature for 20 d. (*n* = 5).

The skin and flesh of ‘Hongro’, which developed greasiness, and ‘Fuji’, which did not, were separated after 20 d at room temperature, and the fatty acids and metabolites between the breeds were compared to analyze the components inducing greasiness. ‘Hongro’ and ‘Fuji’ were stored at low temperatures for the same period, and the fatty acids and untargeted metabolites of their flesh and skin at low and room temperatures were comparatively analyzed using PLS-DA. Principal components 1 and 2 explained 81.9% and 15% of the overall variance, respectively. The pericarp clusters of ‘Hongro’ and ‘Fuji’ differed in the PLS-DA score plot; however, the metabolites present in the flesh showed a similar trend (Figure 3A,B). While the flesh components were clustered on the right, regardless of variety, the skin components were clustered on the left, and divided into upper (‘Hongro’ skin) and lower (‘Fuji’ skin) halves. These differences suggest that the flesh does not differ between varieties, but the skin has distinct metabolites depending on the variety.

Analysis of the two apple varieties identified 10 fatty acids that were more prevalent in the peel than in the flesh (Figures S2 and S3). Palmitic acid (16:0), stearic acid (18:0), oleic acid (18:1), linoleic acid (18:2), α-linolenic acid (18:3), and arachidic acid (20:0) were found in both flesh and skin of both varieties. Lauric acid (12:0), myristic acid (14:0), heneicosanoic acid (21:0), and lignoceric acid (24:0) were also detected in the fruit skin. The fatty-acid content of the fruit skin was 5–10 times that of the flesh (Table 1).

According to the comparison of the fatty-acid content in fruit skin based on variety and storage temperature, the fatty acids present in abundance in the room-temperature-stored ‘Hongro’ skin were lauric acid, myristic acid, arachidic acid, and heneicosanoic acid. Palmitic acid, oleic acid, linoleic acid, and α-linolenic acid were abundantly present in ‘Fuji’ skin. After 20 d of room-temperature and low-temperature storage, ‘Hongro’ had similar amounts of unsaturated and saturated fatty acids, whereas ‘Fuji’ had approximately twice the content of unsaturated fatty acids at low temperatures. Similar results were observed in apples stored at room temperature.

Interestingly, while greasiness occurred at room temperature, it did not occur in low-temperature-stored ‘Hongro’, indicating that other components, including fatty acid, played a role in greasiness in ‘Hongro’. In addition, compared to ‘Hongro’, which was extremely greasy, the fatty-acid content of ‘Fuji’ was higher in apples stored at both low and room temperatures. Very-long-chain fatty acids (VLCFAs) are the main precursors for cuticular wax biosynthesis [23,24,25]. Elongation of C16 and C18 fatty acids results in epidermal wax synthesis. These VLCFAs produce alcohols and esters through the alcohol-forming pathway and aldehydes, alkanes, and secondary alcohols through the alkane-forming pathway [4,5,26].

**Figure 4:**
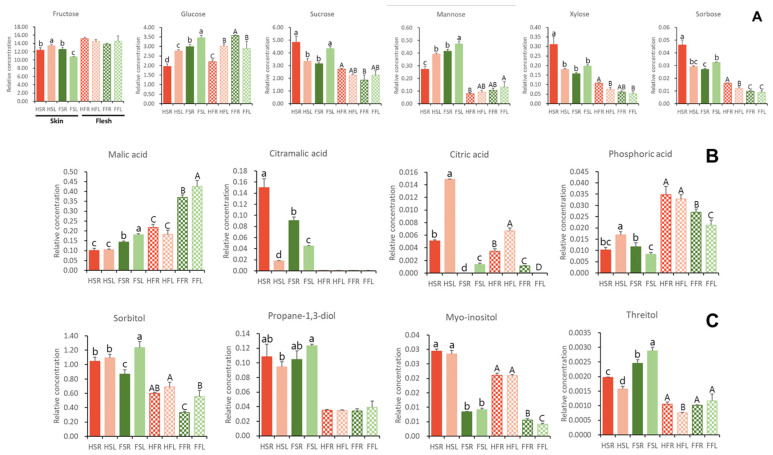
Comparison of metabolome profiles of apples (‘Hongro’ vs. ‘Fuji’). Values are expressed as the means ± standard deviations. Different lowercase letters (between skins) and uppercase letters (between fleshes) indicate significant differences at *p* < 0.05 based on Duncan’s multiple range test. (**A**) Soluble sugars, (**B**) organic acids, and (**C**) sugar alcohols. FFL, ‘Fuji’ flesh stored at low temperatures for 1 month; FFR, ‘Fuji’ flesh stored at room temperature for 20 d; FSL, ‘Fuji’ skin stored at low temperatures for 1 month; FSR, ‘Fuji’ skin stored at room temperature for 20 d; HFL, ‘Hongro’ flesh stored at low temperatures for 1 month; HFR, ‘Hongro’ flesh stored at room temperature for 20 d; HSL, ‘Hongro’ skin stored at low temperatures for 1 month; and HSR, ‘Hongro’ skin stored at room temperature for 20 d. (*n* = 5).

The untargeted polar metabolites of ‘Hongro’ and ‘Fuji’ were broadly classified into sugars, acids, and alcohols. These metabolites differed between varieties, with distinct characteristics between the flesh and skin (Figure S4). The sugars found in the two apple varieties were fructose, glucose, sucrose, mannose, xylose, and sorbose, with fructose, sucrose, and glucose having the highest relative content in that order (Figure 4A). Sugars with low concentrations, such as mannose, xylose, and sorbose, were found in higher concentrations in the skin than in the flesh. The highest relative concentrations of xylose and sorbose were found in ‘Hongro’ fruit skin stored at room temperature.

Malic acid was the primary acid in apples, with citramalic acid and citric acid also present (Figure 4B). Malic acid was abundant in fruit skin, and its content was higher in ‘Fuji’ than in ‘Hongro’, whereas citramalic acid was abundant in fruit skin. Their relative concentration was low in low-temperature-stored ‘Fuji’ and ‘Hongro’. However, when stored at room temperature, their relative concentration was high, with ‘Hongro’ showing more greasiness and the highest relative concentration value (*p* < 0.05). The citric acid content in apples is typically low, and unlike malic acid, the relative concentration of citric acid was found to be high in ‘Hongro’.

The alcohols found in apples were sorbitol, propane-1,3-diol, myo-inositol, and threitol, with sorbitol being the most abundant. The apple skin contained more alcohol than the flesh (Figure 4C). Myo-inositol was found in high concentrations in ‘Hongro’, and its aroma was strong in the pericarp (*p* < 0.05) [27]. Greasiness occurred specifically in the ‘Hongro’ variety stored at room temperature. Furthermore, distinct differences in metabolites present in fruit skin caused greasiness.

### 3.2. Effect of Low-Temperature Storage on ‘Hongro’ Greasiness

**Figure 5:**
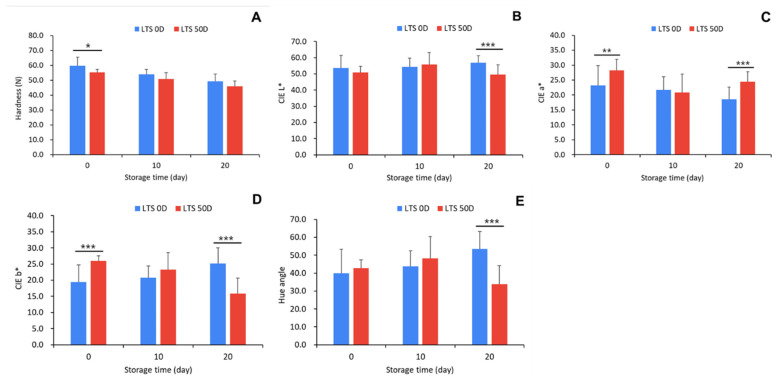
Changes in firmness (**A**), CIE L* (**B**), CIE a* (**C**), CIE b* (**D**), and hue angle (**E**) of ‘Hongro’ apples during storage at room temperature (20 °C). Apples were stored at low temperatures for 0 d (LTS 0D) and 50 d (LTS 50D) before being transferred to room temperature. Values are expressed as the means ± standard deviations. * represents significant differences, * *p* < 0.05, ** *p* < 0.01, and *** *p* < 0.001. (*n* = 6).

To investigate whether greasiness differs depending on the storage period of ‘Hongro’, the apples were harvested in September, the optimal harvest time, and stored immediately after harvest (LTS 0D) or for 50 days (LTS 50D) at low temperatures. After 20 d of storage at 20 °C, the phytochemical properties and greasiness of ‘Hongro’ were investigated at low temperatures. Firmness was significantly higher in apples at the beginning of harvest (*p* < 0.05) (Figure 5A). However, as the room temperature storage period elapsed, there was no significant difference between the LTS 0D and LTS 50D treatments. CIE L* values did not differ between LTS 0D and LTS 50D after 10 d of room temperature storage but differed significantly after 20 d (*p* < 0.001). The skin lightness of apples treated with LTS 50D decreased (Figure 5B). The CIE a* value was significantly higher in LTS 0D (Figure 5C), suggesting differences in ‘Hongro’ skin characteristics from the initial harvest. The CIE b* value of LTS 0D increased during room-temperature storage, while that of LTS 50D decreased (Figure 5D). This change in CIE a* and b* affected the hue angle; after 20 d of room-temperature storage, the hue-angle value of LTS 50D was below 40, a highly significant difference compared to LTS 0D (*p* < 0.001) (Figure 5E), indicating that the fruit skin color is more red. The ripening of the apples progressed when stored for 50 d at low temperatures, presumably because the ripening progressed further after being moved to room temperature.

**Figure 6:**
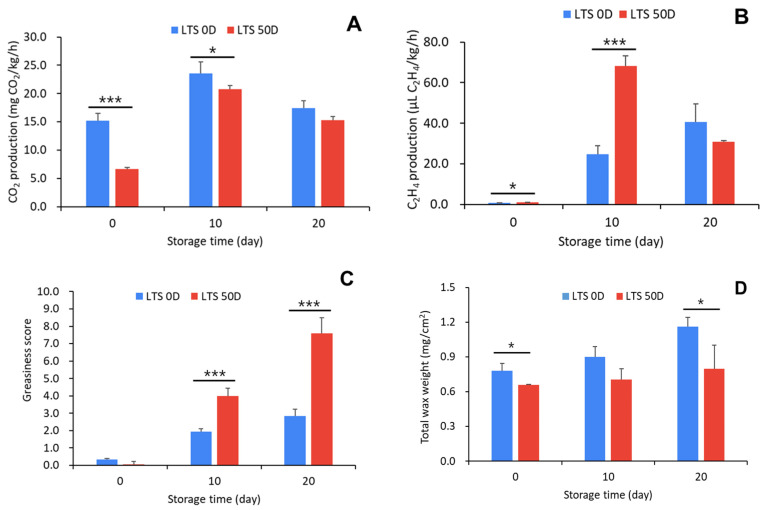
Changes in CO_2_ production (**A**), C_2_H_4_ production (**B**), greasiness score (**C**), and total wax weight (**D**) of ‘Hongro’ apples during storage at room temperature (20 °C). Apples were stored at low temperatures for 0 d (LTS 0D) and 50 d (LTS 50D) before being transferred to room temperature. Values are expressed as the means ± standard deviations. * represents significant differences, * *p* < 0.05 and *** *p* < 0.001. (*n* = 6).

Apple respiration rate was significantly higher in the LTS 0D treatment group than in the LTS 50D treatment group (*p* < 0.001) (Figure 6A). However, as the room-temperature storage period elapsed, the respiration-rate difference decreased. However, there was a significant difference (*p* < 0.05) until 10 d of storage but not after 20 d. In contrast, the amount of ethylene production was significantly (*p* < 0.05) high in LTS 50D-treated ‘Hongro’ from the beginning of room-temperature storage, and a highly significant difference was observed after 10 d of storage at room temperature (*p* < 0.001). As time passed, there was no significant difference in respiration (Figure 6B). This difference in ethylene also showed a similar trend in greasiness in ‘Hongro’ pericarp. Greasiness was more common when the LTS 50D group was stored at room temperature than when the LTS 0D group was showing a significant difference (*p* < 0.001) (Figure 6C). GS was calculated after 10 d of storage, and it differed depending on the storage period. The GS of the LTS 0D treatment group was <3 even after the end of room-temperature storage, which is below the greasiness-acceptability range for consumers. The GS of the LTS 50D treatment group was >4 after 10 d of room-temperature storage. After 20 d, the difference grew larger depending on storage temperature and period (*p* < 0.001).

LTS 50D had a high greasiness score of >8 and was significantly different from LTS 0D (*p* < 0.001). During apple storage, the development of greasy and waxy cuticles is related to storage temperature, storage period, and changes in the wax composition of stored apples [5,12]. The accumulation of wax components causes the wax to change from a solid to liquid phase, and ethylene tends to accelerate this change [12]. The total wax weight of LTS 0D was greater than that of LTS 50D, and the increase in wax weight showed a slight increase as the storage period elapsed (Figure 6D). Interestingly, considering that the total wax weight is significant in LTS 0D, where GS is relatively low, greasiness may be caused by a change in phase rather than an increase in the wax layer.

Greasiness in apple skin develops with apple ripening and increases when well-ripened apples are harvested and stored [4,10]. Apple varieties with well-developed cuticular wax can minimize moisture loss and be more effective for long-term storage than varieties with a thin wax layer [7,26]. Furthermore, the wax layer in plants protects the plant from pathogen attack and allows foreign substances such as pathogens and dust to be easily removed [6,26,28]. Changes in apple cuticular-wax composition result in pathogen infection [26].

When greasiness occurred, the composition of the wax layer of apple skin changed. The main components of ‘Hongro’ wax were alkanes, alcohols, aldehydes, fatty acids, esters, terpenes, and terpenoids, with alkanes, alcohols, and terpenoids having the highest content in that order (Table 2). Wax composition changes revealed an increase in content in the LTS 50D treatment group compared to the LTS 0D treatment group. The content increased further at room temperature after being stored at low temperature for 50 d (*p* < 0.05).

The content of alcohols, alkanes, and esters in LTS 50D ‘Hongro’ increased and tended to increase as the period elapsed after being moved to room temperature. This is similar to the increase in greasiness as exposure time to room temperature elapses. The wax layer of ‘Hongro’ fruit skin contained heneicosane (C21), docosane (C22), pentacosane (C25), heptacosane (C27), nonacosane (C29), triacontane (C30), and hentriacontane (C31). C29 was the most abundant, followed by C27 (Table 3). Furthermore, the period of low-temperature storage affected the increase in alkane. C29 was 9.77 μg cm^−2^ after harvest but increased to 14.45 μg cm^−2^ during 50 d of low-temperature storage. When exposed to room temperature, LTS 0D and LTS 50D were both 13.17 μg cm^−2^, which increased to 18.87 μg cm^−2^, showing a significant difference (*p* < 0.05). Except for C27, all alkanes followed this trend.

**Figure 7:**
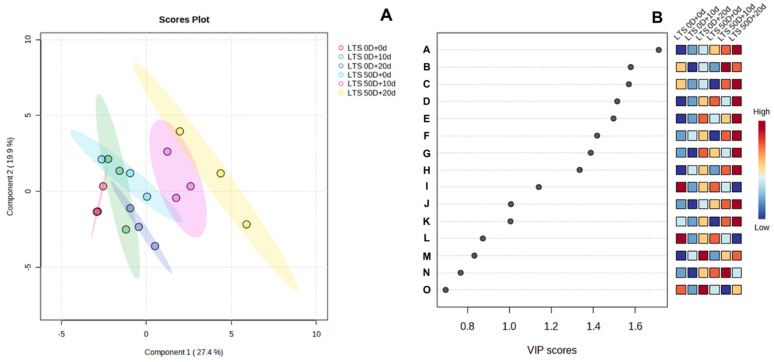
Comparison of the non-polar metabolome of ‘Hongro’ apple during storage at room temperature (20 °C). Apples were stored at low temperatures for 0 d (LTS 0D) and 50 d (LTS 50D) before being transferred to room temperature. (**A**) Score plot and (**B**) VIP score obtained using partial least squares discriminant analysis. A, trans,trans-9,12-octadecadienoic acid, propyl ester; B, 2-oleoylglycerol; C, decanedioic acid, bis(2-ethylhexyl) ester; D, nonacosan-10-ol; E, α-farnesene; F, pentyl linoleate; G, C29; H, 3,7,11-trimethyldodeca-2,6,10-trien-1-yl palmitate; I, 1-hexacosanol; J, 1-monopalmitin; K, erythrodiol; L, 1-octacosanol; M, (2E,6E)-3,7,11-trimethyldodeca-2,6,10-trien-1-yl dodecanoate; N, docosanoic acid, docosyl ester; and O, nonacosanal. (*n* = 5).

The LTD 0D and LTD 50D groups were stored at room temperature for 10 and 20 d, and PLS-DA analysis was performed on ‘Hongro’ fruit skin’s untargeted non-polar metabolites (Figure 7). The LTS 0D and LTS 50D clusters differed significantly, particularly in LTS 50D. Clusters were clearly distinguished after 10 and 20 d of room-temperature storage. This difference indicates that ‘Hongro’ has distinct metabolites when stored for an extended period at low temperatures rather than immediately after harvest. The difference was particularly evident when the room-temperature storage period was prolonged after long-term low-temperature storage. Metabolites corresponding to LTS 50D + 20D had high VIP scores for components with VIP scores of 0.8 or higher for significant metabolites according to each treatment (Figure 7B).

Nonacosan-10-ol was the main alcohol found in the wax component of ‘Hongro’ fruit skin. Nonacosan-10-ol increased with ‘Hongro’ greasiness, particularly in the LTS 50D treatment group. The relative nonacosan-10-ol content was significantly high (*p* < 0.05) (Figure S5). Nonacosan-10-ol is the main wax component of ‘Jonagold,’ which often causes greasiness during room-temperature storage. However, it is not found in the ‘Elstar’ variety [29]. Nonacosan-10-ol directly affects greasiness development in Jonagold during shelf life [29]. Herein, nonacosan-10-ol and nonacosane showed the most significant increase in relative concentration during room-temperature storage. According to previous research, nonacosane and nonacosan-10-ol are the main components of apple epidermal wax and contribute to its structure [29,30].

The PLS-DA VIP score revealed that, among the significant components that tended to increase during room-temperature storage in the LTS 0D and LTS 50D groups, the components with a VIP score of 1.4 or higher were ester-type pentyl linoleate and terpenes type α-farnesene (Figure 7 and Figure S5). Pentyl linoleate and α-farnesene were the most abundant, particularly when greasiness occurred after 20 d at room temperature. The relative concentration was lower than that of other components present in the wax layer, but it was markedly increased due to its accumulation. This confirms the role of pentyl linoleate and α-farnesene in the development of greasiness. According to previous research, there was no difference in the appearance of ‘Red Delicious’, ‘Jonagold’, and ‘Cripps Pink’ during harvest. However, the fluid wax content of ‘Jonagold’ and ‘Cripps Pink’ fruit skin increased during storage. The pericarp’s greasiness is caused by a phase transition from the solid crystal form to the liquid amorphous state as the wax slowly melts [5].

The wax composition changes with greasiness in the fruit skin, with an increase in esters and short-chain alcohol [4]. Linoleate ester dominates in the ‘Royal Gala’ depending on the time when greasiness occurs. Furthermore, the fatty-acid ester of farnesyl increases in ‘Cripps Pink’ with greasiness [31]. In this study, the relative concentration of farnesyl laurate increased with time at room temperature, but there was no difference during low-temperature storage (Figure S5); however, this result cannot explain the reason for greasiness. This result differs from the significant difference in GS occurrence between the LTS 50D and LTS 0D treatment groups (Figure 6C). These findings indicate that esters increase with wax phase conversion when greasiness occurs [5,11,32].

Similarly, α-farnesene was not detected in early harvested ‘Royal Gala’ but increased to 5.5 μg cm^−2^ in late-harvested apple skin. Additionally, 1-MCP inhibited α-farnesene and reduced greasiness [10,11]. α-Farnesene exists in liquid form at room temperature and has been proposed as a critical compound causing greasiness [11].

Environmental and genetic factors influence cuticular wax biosynthesis and deposition [19,33]. Environmental factors include humidity, light, temperature, and pathogens. Under drought stress, plants accumulate cuticular wax in the epidermis, which manifests as shiny, dark-green leaves and enhances drought tolerance [34]. Cuticular wax in apples is directly affected by light and temperature during postharvest storage [3]. Heat treatment at 38 °C can limit Ca^2+^ absorption by preventing the formation of deep cracks in the epicuticular wax of the ‘Golden Delicious’ apple skin [35]. Low-temperature treatment promotes the development and thickness of cuticular wax [36]. Furthermore, UV-B (280–320 nm) radiation treatment increases the total cuticular-wax content and affects wax composition [37].

In addition to these environmental factors, genetic factors influence cuticular wax biosynthesis and deposition. ‘Jonagold’ and ‘Cripps Pink’ apple varieties are the most prone to greasiness among those grown in the United States. ‘Jonagold’ was bred by crossing ‘Golden Delicious’ and ‘Jonathan’, and ‘Cripps Pink’ was bred by crossing ‘Lady Williams’ and ‘Golden Delicious’ [23]. Both varieties with a high occurrence of greasiness used ‘Golden Delicious’ as the maternal or paternal parent. ‘Hongro’ was also bred using ‘Golden Delicious’ as a paternal parent [2], which may have had a genetic influence on ‘Hongro’’s greasiness.

## 4. Conclusions

In this study, we compared the physiological characteristics of two apple varieties, ‘Hongro’ and ‘Fuji’. We discovered greasiness, primarily in ‘Hongro’ apple skin, after 20 d at 20 °C. Ethylene triggers greasiness, and its production rapidly increases before greasiness occurs. The metabolites in ‘Hongro’ and ‘Fuji’ fruit skin differed noticeably. Arachidic acid and lauric acid contents were high in ‘Hongro’ pericarp. Myo-inositol and arabinose showed the highest VIP score and differentiated characteristics from ‘Fuji’. Greasiness increased, particularly in ‘Hongro’, as the low-temperature storage period increased. Ethylene production was increased in ‘Hongro’ stored at room temperature after being stored at low temperature for an extended period, which was directly related to greasiness development. After 20 d at room temperature, the main components involved in the greasiness of ‘Hongro’ are nonacosane, nonacosan-10-ol, pentyl linoleate, and ɑ-farnesene. Furthermore, these ‘Hongro’ characteristics appeared to have been genetically influenced by ‘Golden Delicious,’ the paternal parent used during breeding.

## Figures and Tables

**Table 1:** Comparison of fatty acids between ‘Hongro’ and ‘Fuji’ apples.

Variety/ Storage Temp	Fatty Acid Content (mg/g DW)		
	Saturated Fatty Acid	Unsaturated Fatty Acid	Total Fatty Acid
HSR	4.15 ± 0.18 ^b^	3.58 ± 0.09 ^d^	7.73 ± 0.27 ^c^
HSL	4.16 ± 0.19 ^b^	3.90 ± 0.12 ^c^	8.06 ± 0.31 ^c^
FSR	3.92 ± 0.10 ^b^	7.40 ± 0.20 ^b^	11.32 ± 0.30 ^b^
FSL	4.54 ± 0.13 ^a^	9.14 ± 0.12 ^a^	13.68 ± 0.24 ^a^

The values represent mean ± SD, and different superscript letters in each column represent significant differences (*p* < 0.05) based on Duncan’s multiple range test. FSL, ‘Fuji’ skin stored at low temperatures for 1 month; FSR, ‘Fuji’ skin stored at room temperature for 20 d; HSL, ‘Hongro’ skin stored at low temperatures for 1 month; HSR, ‘Hongro’ skin stored at room temperature for 20 d. (*n* = 5).

**Table 2:** Changes in the non-polar metabolome of ‘Hongro’ apples during storage at room temperature (20 °C). Apples were stored at low temperatures for 0 d (LTS 0D) and 50 d (LTS 50D) before being transferred to room temperature.

Treatment	Wax Composition (µg/cm^2^)						
	Alcohols	Aldehydes	Alkane	Esters	Fatty Acid	Terpenes	Terpenoids
LTS 0D + 0 d	9.51 ± 0.54 ^b^	0.66 ± 0.12 ^ab^	11.27 ± 0.43 ^b^	4.84 ± 0.58 ^b^	8.05 ± 0.25 ^c^	0.65 ± 0.03 ^c^	11.40 ± 1.89 ^ab^
LTS 0D + 10 d	10.62 ± 3.22 ^b^	0.47 ± 0.16 ^ab^	12.68 ± 3.68 ^b^	5.71 ± 1.36 ^b^	10.54 ± 1.25 ^ab^	2.50 ± 0.91 ^ab^	8.46 ± 2.50 ^b^
LTS 0D + 20 d	12.62 ± 1.62 ^ab^	0.77 ± 0.15 ^a^	15.09 ± 1.39 ^ab^	9.15 ± 2.16 ^a^	9.54 ± 0.97 ^bc^	3.67 ± 0.90 ^a^	10.70 ± 0.84 ^ab^
LTS 50D + 0 d	15.06 ± 4.51 ^ab^	0.52 ± 0.13 ^ab^	16.27 ± 4.55 ^ab^	5.77 ± 1.09 ^b^	10.13 ± 0.93 ^ab^	0.65 ± 0.17 ^c^	15.32 ± 5.23 ^a^
LTS 50D + 10 d	14.51 ± 2.27 ^ab^	0.35 ± 0.31 ^b^	16.22 ± 2.47 ^ab^	8.99 ± 1.29 ^a^	11.57 ± 1.40 ^a^	2.15 ± 0.76 ^b^	11.45 ± 1.86 ^ab^
LTS 50D + 20 d	17.65 ± 3.41 ^a^	0.70 ± 0.10 ^a^	20.79 ± 3.48 ^a^	11.84 ± 2.27 ^a^	11.01 ± 0.24 ^ab^	2.38 ± 0.83 ^ab^	14.53 ± 3.29 ^a^

The values represent mean ± SD, and different superscript letters in each column represent significant differences (*p* < 0.05) based on Duncan’s multiple range test.

**Table 3:** Changes in alkane content of ‘Hongro’ apple during storage at room temperature (20 °C). Apples were stored at low temperatures for 0 d (LTS 0D) and 50 d (LTS 50D) before being transferred to room temperature.

	Shelf Life (Day)	Alkane Content (µg/cm^3^)							
		C21	C22	C25	C27	C29	C30	C31	Total Alkane
LTS 0D	0	Nd	0.08 ± 0.02 ^b^	0.18 ± 0.01 ^b^	1.50 ± 0.08 ^a^	9.77 ± 0.35 ^b^	0.12 ± 0.02 ^b^	0.25 ± 0.01 ^c^	11.92 ± 0.42 ^b^
	10	Nd	0.09 ± 0.01 ^ab^	0.23 ± 0.01 ^ab^	1.68 ± 0.68 ^a^	11.02 ± 2.97 ^b^	0.16 ± 0.05 ^ab^	0.29 ± 0.03 ^c^	13.60 ± 3.92 ^b^
	20	0.12 ± 0.02 ^b^	0.12 ± 0.02 ^ab^	0.26 ± 0.05 ^ab^	1.94 ± 0.37 ^a^	13.17 ± 1.06 ^b^	0.18 ± 0.02 ^ab^	0.31 ± 0.00 ^bc^	16.10 ± 1.41 ^ab^
LTS 50D	0	0.09 ± 0.01 ^b^	0.00 ± 0.00 ^c^	0.22 ± 0.04 ^b^	1.81 ± 0.51 ^a^	14.45 ± 4.09 ^ab^	0.18 ± 0.06 ^ab^	0.42 ± 0.12 ^ab^	17.25 ± 2.91 ^ab^
	10	0.14 ± 0.01 ^b^	0.11 ± 0.01 ^ab^	0.22 ± 0.03 ^b^	1.70 ± 0.41 ^a^	14.52 ± 2.07 ^ab^	0.22 ± 0.02 ^a^	0.42 ± 0.04 ^ab^	17.38 ± 2.44 ^ab^
	20	0.32 ± 0.00 ^a^	0.14 ± 0.05 ^a^	0.33 ± 0.03 ^a^	1.92 ± 0.47 ^a^	18.87 ± 3.02 ^a^	0.22 ± 0.05 ^a^	0.48 ± 0.08 ^a^	22.32 ± 2.02 ^a^

The values represent mean ± SD, and different superscript letters in each column represent significant differences (*p* < 0.05) based on Duncan’s multiple range test. (*n* = 5).

## Data Availability

The datasets generated during the current study are publicly available from the corresponding author upon request.

## Supplementary Materials

The following supporting information can be downloaded at https://www.mdpi.com/article/10.3390/foods12224088/s1: Figure S1: Changes in CIE L* (**a**), CIE b* (**b**), CIE a* (**c**), chroma (**d**), and hue angle (**e**) of ‘Hongro’ and ‘Fuji’ apples during room-temperature storage (20 °C); Figure S2: Comparison of fatty acids between ‘Hongro’ and ‘Fuji’ apples; Figure S3: Comparison of fatty acids between ‘Hongro’ and ‘Fuji’ apples with VIP score obtained using partial least squares discriminant analysis; Figure S4: Comparison of polar metabolome in ‘Hongro’ and ‘Fuji’ apples; Figure S5: Changes in nonacosan-10-ol, nonacosane, α-farnesene, pentyl linoleate, and farnesyl laurate of ‘Hongro’ apples during room-temperature storage (20 °C).
