# Supplementary material for: Comparative Analysis of Metabolites of ‘Hongro’ Apple Greasiness in Response to Temperature
Source: Foods. 2023 Nov 10;12(22):4088. doi: 10.3390/foods12224088 (PMC10670088; doi:10.3390/foods12224088)
Supplement: Supplementary file 1 [file foods-12-04088-s001.zip › foods-2691031-supplementary.pdf]

Supplementary Materials

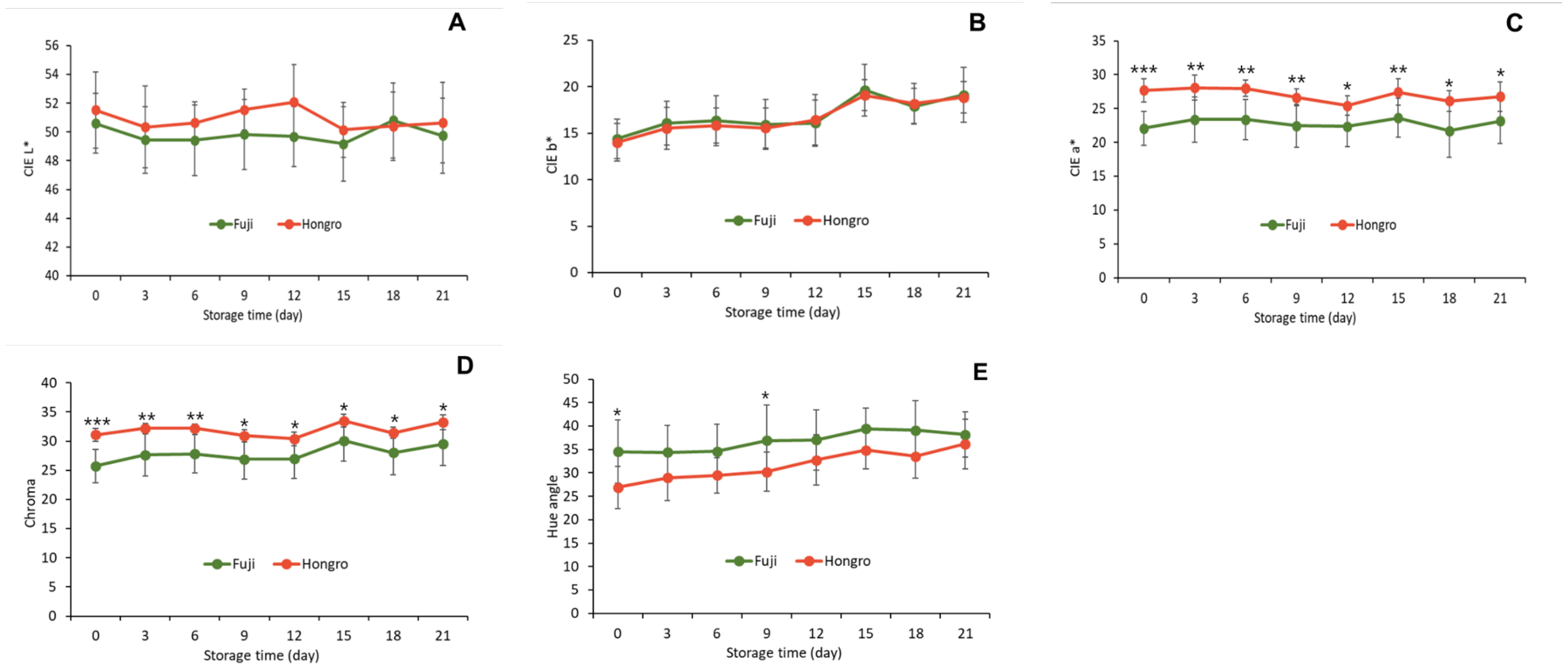

Figure S1. Changes in CIE L* (**a**), CIE b* (**b**), CIE a* (**c**), chroma (**d**), and hue angle (**e**) of Hongro and Fuji apples during room-temperature storage (20 °C). Values are expressed as the means ± standard deviations. * represents significant differences, * $P < 0.05$, ** $P < 0.01$, and $P < 0.001$.

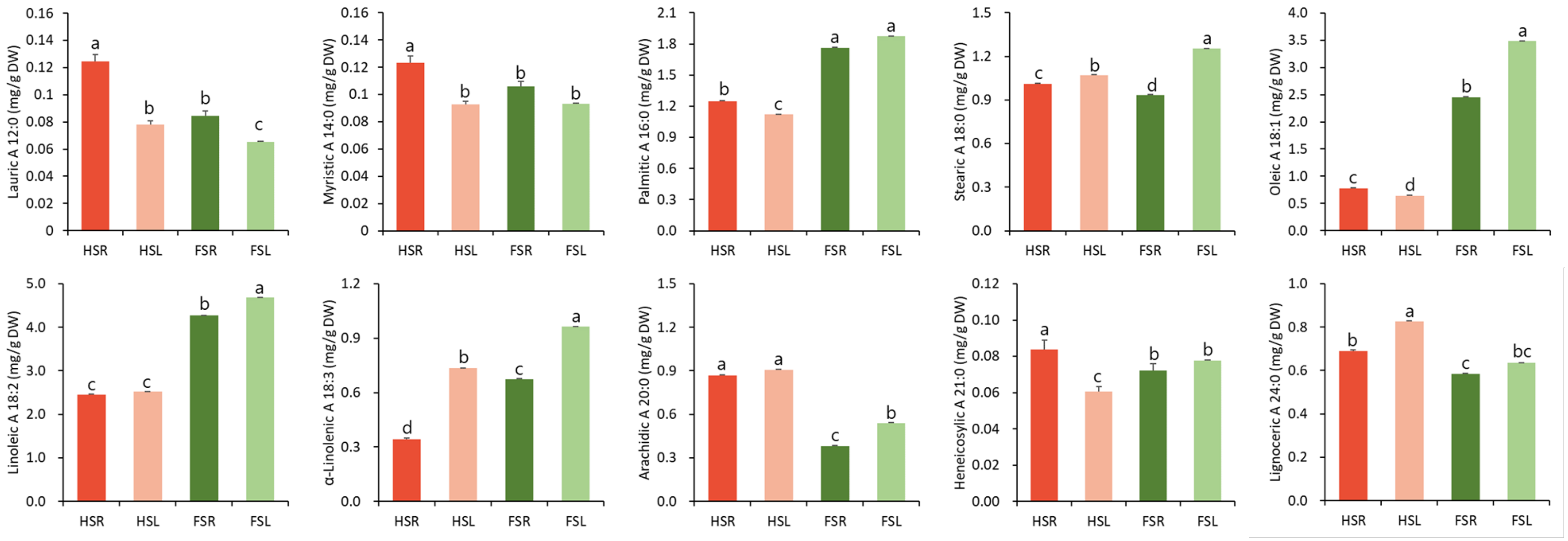

Figure S2. Comparison of fatty acids between Hongro and Fuji apples. Values are expressed as the means ± standard deviations. Different lowercase letters indicate significant differences at $P < 0.05$ based on Duncan's multiple range test. Abbreviations are FSL, Fuji skin stored at low temperatures for 1 month; FSR, Fuji skin stored at room temperature for 20 d; HFL, Hongro flesh stored at low temperatures for 1 month; and HFR, Hongro flesh stored at room temperature for 20 d.

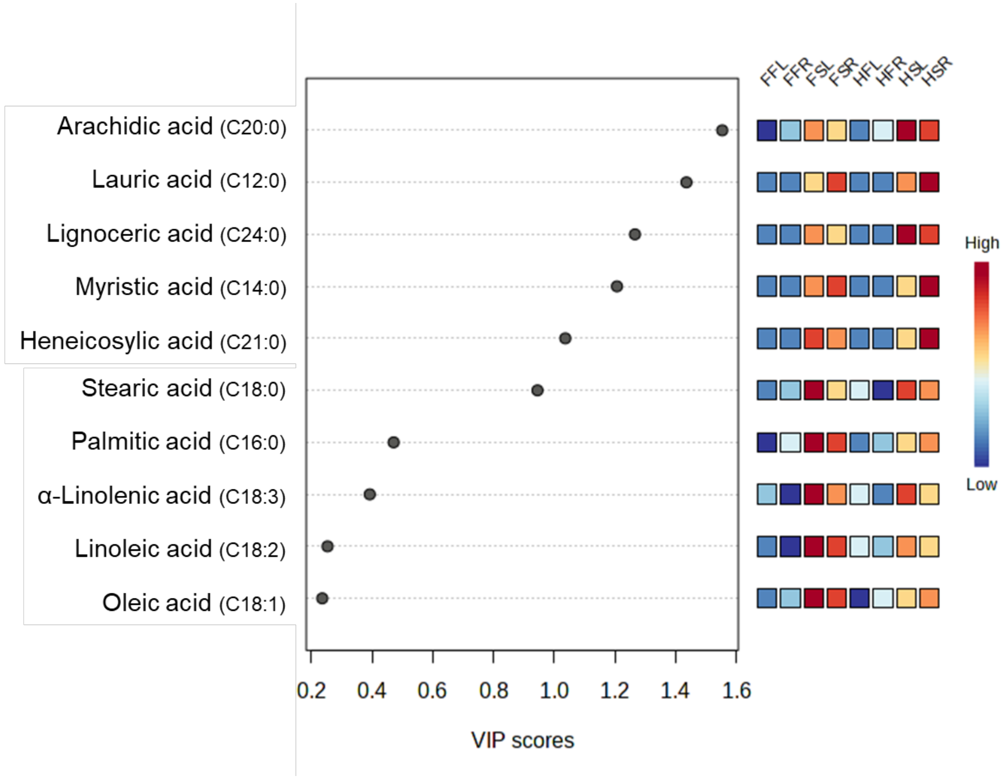

Figure S3. Comparison of fatty acids between Hongro and Fuji apples with VIP score obtained using partial least squares discriminant analysis. Values are expressed as the means ± standard deviations. FFL, Fuji flesh stored at low temperatures for 1 month; FFR, Fuji flesh stored at room temperature for 20 d; FSL, Fuji skin stored at low temperatures for 1 month; FSR, Fuji skin stored at room temperature for 20 d; HFL, Hongro flesh stored at low temperatures for 1 month; HFR, Hongro flesh stored at room temperature for 20 d; HSL, Hongro skin stored at low temperatures for 1 month; and HSR, Hongro skin stored at room temperature for 20 d.

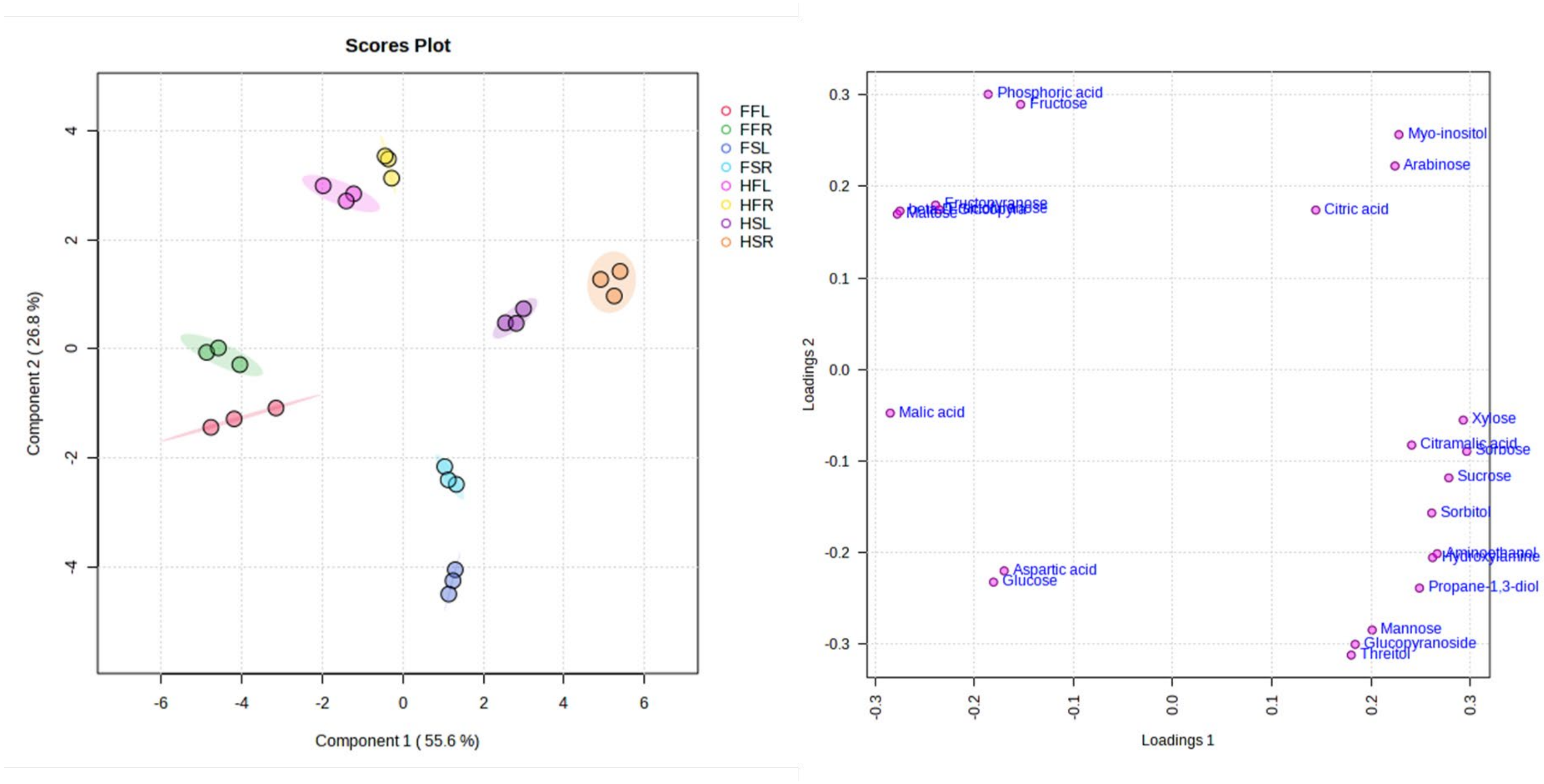

Figure S4. Comparison of polar metabolome in Hongro and Fuji apples. (**a**) Score plot and (**b**) loading plot obtained using partial least squares discriminant analysis of fatty acids in skin and flesh of both apple cultivars. Values are expressed as the means ± standard deviations. FFL, Fuji flesh stored at low temperatures for 1 month; FFR, Fuji flesh stored at room temperature for 20 d; FSL, Fuji skin stored at low temperatures for 1 month; FSR, Fuji skin stored at room temperature for 20 d; HFL, Hongro flesh stored at low temperatures for 1 month; HFR, Hongro flesh stored at room temperature for 20 d; HSL, Hongro skin stored at low temperatures for 1 month; and HSR, Hongro skin stored at room temperature for 20 d.

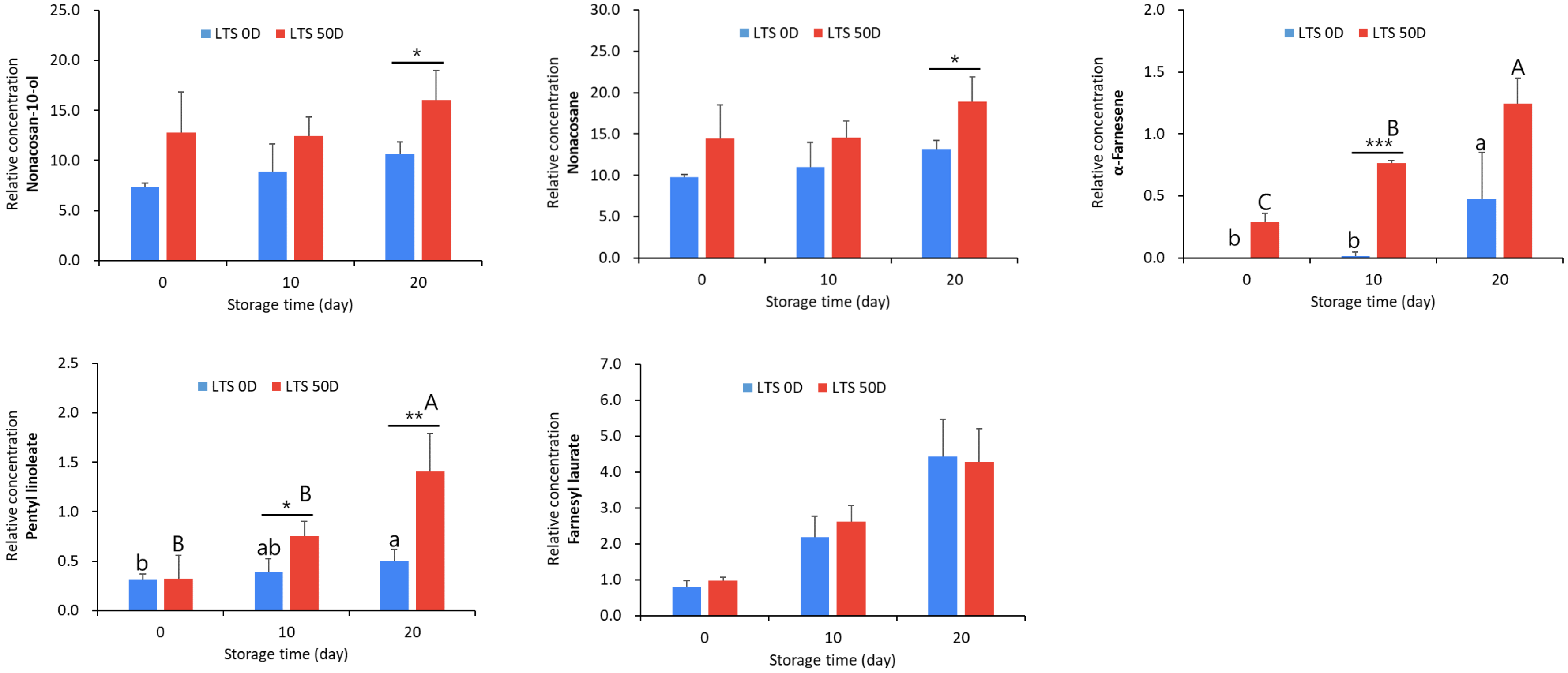

Figure S5. Changes in nonacosan-10-ol, nonacosane, α-farnesene, pentyl linoleate, and farnesyl laurate of Hongro apples during room-temperature storage (20 ℃). Different lowercase letters (LTS 0D) and uppercase letters (LTS 50D) indicate significant differences at $P < 0.05$ based on Duncan's multiple range test. Apples were stored at low temperatures for 0 d (LTS 0D) and 50 days (LTS 50D) before being moved to room temperature. Values are expressed as the means ± standard deviations. * represents significant differences, * $P < 0.05$, ** $P < 0.01$, and *** $P < 0.001$.
